# What Competences Are Required to Deliver Person-Person Behaviour Change Interventions: Development of a Health Behaviour Change Competency Framework

**DOI:** 10.1007/s12529-020-09920-6

**Published:** 2020-07-20

**Authors:** Diane Dixon, Marie Johnston

**Affiliations:** Institute of Applied Health Sciences, School of Medicine, Medical Sciences and Nutrition, University of Aberdeen, Aberdeen, AB25 2ZD Scotland

**Keywords:** Professional competency, Behaviour change interventions, BCTs, Behaviour change techniques, Training

## Abstract

**Background:**

The competence of the person delivering person-to-person behaviour change interventions may influence the effectiveness of the intervention. However, we lack a framework for describing the range of competences involved. The objective of the current work was to develop a competency framework for health behaviour change interventions.

**Method:**

A preliminary framework was developed by two judges rating the relevance of items in the competency framework for cognitive behaviour therapies; adding relevant items from reviews and other competency frameworks; and obtaining feedback from potential users on a draft framework. The Health Behaviour Change Competency Framework (HBCCF) was used to analyse the competency content of smoking cessation manuals.

**Results:**

Judges identified 194 competency items as relevant, which were organised into two domains: foundation (12 competency topics comprising 56 competencies) and behaviour change (12 topics, 54 competencies); several of the 54 and 56 competencies were composed of sub-competencies (84 subcompetencies in total). Smoking cessation manuals included 14 competency topics from the foundation and behaviour change competency domains.

**Conclusion:**

The HBCCF provides a structured method for assessing and reporting competency to deliver behaviour change interventions. It can be applied to assess a practitioner’s competency and training needs and to identify the competencies needed for a particular intervention. To date, it has been used in self-assessments and in developing training programmes. We propose the HBCCF as a practical tool for researchers, employers, and those who design and provide training. We envisage the HBCFF maturing and adapting as evidence that identifies the essential elements required for the effective delivery of behaviour change interventions emerges.

**Electronic supplementary material:**

The online version of this article (10.1007/s12529-020-09920-6) contains supplementary material, which is available to authorized users.

## Introduction

The importance of behaviour in determining health outcomes has led to the development of behaviour change interventions designed to improve outcomes by changing the behaviour of people in general, clinical populations, or healthcare professionals. Behaviour change interventions take many forms, and the competencies required to deliver these different forms of intervention are likely to differ widely; for example, delivering a self-help weight management leaflet requires a lesser level of competency than that required to change dietary and sedentary behaviours in a morbidly obese diabetic patient. Furthermore, behaviour change interventions are delivered by health professionals from a variety of disciplines and in a wide range of settings [1]. Practitioners trained in these disciplines may vary greatly in how competent they are to deliver behaviour change interventions. They have different training and, while there is likely to be some shared competencies over different disciplines, there is no framework for describing these competencies. In this paper, we focus on behaviour change interventions delivered person-to-person to individuals or small groups and outline the pragmatic development of a framework of the competencies involved in their delivery by health or other professionals. Interventions using other, non-personalised, modes of delivery [2, 3] are also effective in achieving behaviour change [4] but require other competences outside the scope of the current paper.

The competency of the person delivering an intervention may, in part, determine its effectiveness. Meta-analyses of behaviour change interventions frequently note heterogeneity in the findings and explore possible sources of this heterogeneity [5–8]. The availability of an agreed taxonomy of behaviour change techniques (BCTs) means the role of BCTs in this heterogeneity can be explored and effective BCTs identified [9]. Unfortunately, there is no standard method of reporting the competency of the persons delivering the intervention and it is therefore difficult to ascertain whether this might also explain at least some of the variance in outcomes from behaviour change interventions. That said, there is evidence that the quality of delivery of behaviour change interventions impinges on their effectiveness; Lorencatto and colleagues found that smoking cessation interventions were more effective if the person delivering the intervention ensured that goal-setting was delivered well, but, while they described effective delivery, they did not offer guidance on how to achieve this quality [10]. Several studies also note that interventions may be more effective when delivered by a particular profession: for example, Hartman-Boyce found that weight loss programmes were more effective if a dietician was involved in delivery [11]. On the other hand, An et al. (2008) found that having more than one profession involved was important for success in smoking cessation [1]. It is plausible that having more than one profession involved ensured a wider range of competencies.

The importance of the competency of the person delivering an intervention is increasingly recognised. The TIDieR checklist for the reporting of interventions [12] requires the description of the theoretical processes employed in an intervention, the behaviour change content, and the multiple requirements for its delivery. TIDieR requires not only a description of the person delivering the intervention but also a description of any specific training given. Behaviour change interventions have been described as having two broad components, behaviour change techniques and form-of-delivery, i.e. the way the intervention is delivered [13, 14], which includes professional competencies such as communication style [15]. The important role of interpersonal competencies in behaviour change interventions is increasingly recognised [16] and some have even suggested that interpersonal style might best be considered a behaviour change technique [17].

Competency-based training and assessment is used by a variety of health professions but some of these competencies may be specific to a particular profession. However, behaviour change interventions are delivered by a wide range of disciplines and we therefore need a framework that works across disciplines. Based on consultation with a wide range of disciplines and with national policy-makers responsible for health-related behaviour change, and on our experience, (a) in research on behaviour change interventions including delivery and evaluation in randomised controlled trials, (b) in practice, both in delivering and in managing staff delivering person-to-person behaviour change interventions, and (c) in policy as advisers to national policy-makers and civil servants tasked with implementing policy, we propose that such a framework might serve the following purposes:

- *Implementation*: More precise reporting of competencies of individuals delivering behaviour change interventions and therefore more accurate implementation of effective behaviour change interventions.
- *Evidence synthesis*: Analysis of the impact of competency on effectiveness of RCTs and in evidence synthesis resulting in better analyses of sources of heterogeneity of results.
- *Competence across behaviours and contexts*: Identification of generic competencies that are relevant for changing a wide range of behaviours to inform the movement of staff competent in delivering behaviour change interventions for one behaviour or context to transfer to delivering behaviour change interventions effectively for a different behaviour or in a different setting.
- *Competencies for behaviour change interventions of different complexity*: Identification of competencies needed for behaviour change interventions of increasing degrees of complexity to allow the possibility that staff with lower levels of competence may deliver simple behaviour change interventions while ensuring that complex interventions are only delivered by more competent staff.
- *Job descriptions*: Specification, by employers, of the competencies required for particular roles and potential employees able to choose jobs matched to their own competencies.
- *Staff selection*: Appointment of staff with appropriate competency to deliver behaviour change interventions through the assessment of the competences of available candidates.
- *Training needs*: Specification of training needs for staff delivering behaviour change interventions so that individuals can find appropriate training opportunities or can by guided to such resources by their career guidance personnel.
- *Training programmes*: Specification of the necessary components of training programmes to ensure that the full range of competences are addressed and at the level of complexity appropriate for the staff being trained.

We therefore aimed to develop a behaviour change intervention competency framework to be used in assessing competency to deliver behaviour change interventions by practitioners working directly with clients and patients. This work was undertaken while the authors were on secondment with the Health Directorate of the Scottish Government. It forms a pragmatic response to the expressed need of the Health Directorates (including policy leads for alcohol, smoking, obesity, and drugs) for such a competency framework. Our aim was to develop a working framework that could be updated as required, e.g. through the inclusion of additional competencies and the removal of unnecessary competencies, as additional evidence of the competencies needed to deliver behaviour change interventions accumulates.

It might appear desirable to conduct a content analysis of competence requirements cited in published reports of behaviour change interventions; this proved impossible as, while these reports sometimes made reference to the disciplines of those delivering the interventions, they made little reference to competences required. We therefore took as our starting point the existing competency framework which was nearest to our requirements of describing competence to deliver behaviour change interventions in a person-to-person context, the CBT competency framework for depression and anxiety disorders [18]. CBT was derived directly from behavioural therapy, where the target was to change behaviour directly. The CBT competency framework reflects this and deals with both personal psychological change and behaviour change in interventions delivered person-to-person. Nevertheless, since the CBT framework was planned for use in interventions where behaviour change may not be required, and its constituent techniques for change have a psychological focus, e.g. Socratic questioning, the structure and focus of the framework required adaptation to be suitable for behaviour change interventions.

The aims of this study were to:

1. Develop a health behaviour change competency framework based on the CBT competency framework.
2. Illustrate the use the framework to analyse the competency content of behaviour change interventions, using smoking cessation manuals as the example.

## Method

The research was conducted as two studies, which matched the two aims.

### Study 1: Development of a Health Behaviour Change Competency Framework Based on the CBT Framework

The development of the framework involved four phases: identifying relevant items from the CBT competency framework; identifying additional items from literature (research reports and training manuals) and experts in the area; structuring the resulting items; and obtaining and incorporating feedback from stakeholders.

i. The CBT competency framework consisted of five competency domains [18]. Two registered health psychologists (DD and MJ) independently classified each of the CBT domains as relevant or not to behaviour change interventions. The individual competencies within each behaviour change intervention relevant domain were then each judged as relevant or not relevant to behaviour change interventions delivered person-to-person, to an individual or in small groups. Agreement was assessed using intraclass correlation coefficient two-way mixed model with measure of consistency, to give the equivalent to Cohen’s weighted kappa [19] and then disagreements were resolved by discussion.
ii. The resulting list of competencies was developed and expanded in an iterative process. Additional competencies relevant to health behaviour change were identified by consulting systematic reviews [7, 20–23] of behaviour change interventions and health psychologists working in the area of behaviour change (members of the British Psychological Society Division of Health Psychology – Scotland). Of particular importance was the need to include competency to deliver behaviour change techniques (BCTs)^1^ [9]. A content analysis of a national competency framework and accompanying training manuals for alcohol brief interventions identified generic professional and basic behaviour change competencies [24, 25].
iii. The list of competencies generated was organised using the CBT competency framework as an outline structure in which each competency item was grouped into a competency *topic*, which in turn were grouped into higher order competency *domains*. The structure was adjusted where the health behaviour change competencies differed from those for CBT.
iv. A preliminary framework (incorporating a preliminary version of the Behavior Change Techniques Taxonomy v1 [9])^1^ was presented for formal input and feedback to a wide range of the potential national policy and practitioner users including the National Scottish Government Department of Health policy leaders for health improvement (including policy leaders for healthy living and screening; obesity; smoking and substance abuse; sexual health; alcohol); the Head of Learning and Improvement for NHS Scotland; Programme Director NHS Education Scotland and the section head of NHS Education Scotland with responsibility for training the workforce to deliver behaviour change; Public Health Consultants responsible for regional delivery of behaviour change interventions from rural and urban health boards; and health and clinical psychology practitioners working across three health boards. In addition, the HBCCF was also presented to a variety of stakeholders (practitioners and training leads from a variety of health boards) and feedback received from them regarding whether the HBCCF would be useful for them, what aspects would be most useful, whether the HBCCF was missing important information, and whether any aspect of the HBCCF was unclear. We also consulted health psychology trainees and health psychology conference attenders (British Psychological Society Division of Health Psychology – Scotland Annual Scientific Conference via a plenary presentation and a structured audience feedback session). Stakeholders consulted were aware that their responses would inform the publication of the HBCCF.
v. To facilitate integration with existing competency frameworks used by UK public sector employees, the competencies described in the HBCCF were mapped across to the Knowledge and Skills Framework [26] (KSF). This was completed by one judge (DD).

### Study 2: Illustrating Use of the Framework to Analyse the Competency Requirements of Behaviour Change Interventions

The HBCCF was used to analyse the competency content of ten smoking cessation manuals used in interventions in the UK and USA and included in a systematic review [22]. Coding was conducted for each competency topic and grouped by competency domain by two coders (MJ and DD) independently. Agreement was assessed using intraclass correlation coefficient.

## Results

### Study 1

The CBT framework is comprised of five competency domains of which, in the first phase, three were judged to be potentially relevant to behaviour change interventions namely, generic therapeutic, basic CBT, and metacompetency domains. Two domains were judged to be not relevant, namely specific behavioural and cognitive therapy techniques and problem-specific competencies. The behavioural and cognitive therapy techniques domain described general therapy competencies relevant to the delivery of CBT, for example, guided discovery and Socratic questioning, applied relaxation, and applied tension. The problem-specific competency domain described competencies required to deliver particular interventions for specific psychological conditions, e.g. Resick model of PTSD [27, 28], Beck’s cognitive therapy for depression [29].

The three domains judged as relevant to behaviour change interventions describe 31 competency areas; 26 were judged to contain competencies relevant to health behaviour change (kappa = 1). The 26 competency areas are comprised of 267 specific competencies of which 152 were identified as relevant (kappa equivalent = 0.74). Phase ii identified an additional 42 specific competencies. Some parts of the structure of the CBT framework were retained and the 194 individual competency items relevant to behaviour change interventions were structured into two domains each with 12 *topic*s (see Table 1). Phase iv feedback did not alter the structure but resulted in rephrasing some items to aid intelligibility for multiple disciplines. The resulting HBCCF consists of two domains: foundation competencies organised in 12 topics with a total of 56 competencies and behaviour change competencies organised in 12 topics with a total of 54 competencies. The 56 and 54 competencies of the foundation and behaviour change competency domains are themselves comprised of sub-competencies that make up the total 194 individual competency items of the HBCCF (see Table 1).

**Table 1:** Foundation and behaviour change domains and topics and number of competencies in each topic

Competency domain topics (number of competencies in each topic)	
Foundation	Behaviour change
F1: Professional and ethical guidelines (7)	BC1: Knowledge of health behaviour and health behaviour problems (9)
F2: Ability to make use of supervision (4)	BC2: Ability to undertake a generic assessment (7)
F3: Knowledge of and ability to work with difference (3)	BC3: Knowledge of a model of behaviour change and the ability to understand and employ the model in practice (4)
F4: Ability to communicate and work with different individuals, groups and communities (1)	BC4: Ability to agree goals for the intervention (2)
F5: Ability to engage client (8)	BC5: Capacity to implement behaviour change models in a flexible but coherent manner (2)
F6: Ability to work with groups of clients (11)	BC6: Capacity to select and skillfully apply the most appropriate behaviour change intervention method (1)
F7: Ability to foster and maintain a good intervention alliance, and to grasp the client’s perspective (8)	BC7: Capacity to implement behaviour change in a manner consonant with its underlying philosophy (6)
F8: Capacity to adapt interventions in response to client feedback (3)	BC8: Ability to structure consultations (5)
F9: Ability to manage expectations of the intervention (3)	BC9: Ability to use measures and self-monitoring to guide behaviour change interventions and to monitor outcome (6)
F 10: Ability to deliver information (4)	BC10: Health behaviour problem solving (9)
F11: Capacity to structure consultations and maintain appropriate pacing (3)	BC11: Capacity to manage obstacles to carrying out behaviour change (1)
F12: Barriers to and facilitators of implementing interventions (1)	BC12: Ability to end the intervention in a planned manner and to plan for long-term maintenance of gains after intervention ends (2)

The full list of competencies within each domain is provided in the Electronic Supplementary Material (Supplementary Table 1) but here, we illustrate the contents of a single foundation and a single behaviour change topic (see Table 2).

**Table 2:** Competencies included in illustrative topics within Foundation and Behaviour Change Competency Domains

Foundation domain	Behaviour change domain
Topic F5: Ability to engage client	Topic BC1: Knowledge of health behaviour and health behaviour change
1. Ability to initiate a discussion about potential health behaviour problems	1. Knowledge of common health behaviour problems during assessment and when carrying out interventions, including knowledge of national guidelines for health behaviours, e.g. alcohol consumption limits, recommended physical activity levels, etc.
2. While maintaining professional boundaries, an ability to show appropriate levels of warmth, concern, confidence and genuineness, matched to client need	2. Knowledge of factors associated with the development and maintenance of health behaviours
3. An ability to engender trust	3. Knowledge of the usual patterns of health behaviour problems
4. An ability to develop rapport	4. Knowledge of the ways in which health behaviour problems can impact on health and functioning
5. An ability to convey an appropriate level of confidence and competence	5. Knowledge of the usual knowledge and misinformation that people may have about health behaviour problems
6. An ability to avoid negative interpersonal behaviours (such as impatience, aloofness, or insincerity)	6. Knowledge of main terms and concepts used in epidemiology and the basis of calculations related to these terms
7. An ability to adjust the level and structure of the session to the client’s needs	7. Knowledge of different models, principles and approaches to preventing risk and threats to population health
8. An ability to adapt personal style so that it meshes with that of the client	8. Knowledge of different models, principles and approaches to improving the health of individuals

In order for the HBCCF to be useful to UK public sector employers, in phase v, the competencies were mapped to the Knowledge and Skills Framework (KSF), which they use. Table 3 shows a summary of the mapping between the HBCCF and the KSF. The detailed mapping of each HBCCF competency to the KSF is contained in the Electronic Supplementary Material (Supplementary Table 1).

**Table 3:** Summary of the HBCCF competency topics matched to knowledge and skills framework competency dimensions

KSF dimension sub-dimension	HBCCF competency topics	
	Foundation	Behaviour change
Core dimension		
Communication	F1-F10	BC2, BC7, BC8, BC10, BC11
Personal and people development	F2	na
Service improvement	F1, F2	na
Quality	F1	na
Equality and diversity	F3	na
Health and wellbeing dimension		
Protection of health and wellbeing	F1	na
Enablement to address health and wellbeing needs	F8	BC4, BC7, BC8, BC10
Assessment and treatment planning	F12	BC1-BC3, BC5, BC6, BC8-BC11
Interventions and treatments	F3, F7-F11	BC1, BC4, BC5, BC6-BC10, BC12
Information and knowledge dimension		
Information collection & analysis	F1	na
Knowledge and information resources	F2	na

*na*, KSF sub-dimension not applicable to this HBCC competency domain

Study 1 resulted in the Health Behaviour Change Competency Framework (HBCCF) which was made available for use in the Scottish National Health Service including being published on the Government website http://www.healthscotland.com/documents/4877.aspx.^2^

### Study 2

The competency topics identified in the behavioural support for smoking cessation manuals are shown in Table 4. Kappa for agreement was 0.65. Each manual contains a range of competencies with interventions that delivered individual behavioural support describing more competencies than the others.

**Table 4:** Competency topics from the Foundation and Behaviour Change domains used in smoking cessation interventions

Type of intervention	Domain Topic; *number of times* competencies within each topic are listed in Michie et al. (2011)	
	Foundation	Behaviour change
Individual behavioural support	F1 Knowledge of professional and ethical guidelines; *9* F2 Ability to make use of supervision; *1* F3 Knowledge of and ability to work with difference; *1* F5 Ability to engage client; *1* F7 Ability to foster and maintain a good intervention alliance; *3* F9 Ability to manage expectations; *1* F10 Ability to deliver information; *8*	BC1 Knowledge of health behaviour and health behaviour change; *7* BC2 Ability to take a generic assessment; *11* BC3 Knowledge of models of behaviour change and ability to use them in practice; *1* BC6 Capacity to select and skilfully apply the most appropriate behaviour change intervention method; *1* BC8 Capacity to select and apply the most appropriate intervention method; *1* BC9 Ability to use measures and self-monitoring; *2*
Cochrane review of trials of individual support	F7 Ability to foster and maintain a good intervention alliance; *1* F10 Ability to deliver information; *3*	BC2 Ability to take a generic assessment; *7* BC6 Capacity to select and skilfully apply the most appropriate behaviour change intervention method; *1* BC9 Ability to use measures and self-monitoring; *1*
Group-based behavioural support	F1 Knowledge of professional and ethical guidelines; *1* F6 Ability to work with groups of clients; *10* F10 ability to deliver information; *2*	BC2 Ability to undertake a generic assessment; *3* BC3 Knowledge of models of behaviour change and ability to use them in practice; *1* BC9 Ability to use measures and self-monitoring; *1*
Cochrane review of trials of group-based support	F6 Ability to work with groups of clients; *3* F10 Ability to deliver information; *1*	

## Discussion

The work we describe here has developed and illustrated the use of a HBCCF.

As outlined in the “Introduction” section, there are many reasons for needing a competency framework for the delivery of behaviour change interventions for both research and practical applications. Nevertheless, there is no agreed method of developing such a framework. As a starting point, we used a well-developed framework, based on evidence of effectiveness, which we considered to be the most closely related to our current aims as it addressed changes in behaviour and thinking required to achieve personal change. This was confirmed in the first phase of study 1, where there was complete agreement on the initial judgement carried out at the level of labels for groups of related individual competencies. This judgement task was very straightforward because the activities were rather obviously relevant to the delivery of behaviour change interventions, e.g. ‘knowledge of, and ability to operate within, professional and ethical guidelines’ or were very obviously not, e.g. ‘applied tension and applied relaxation’, i.e. competencies that were very much CBT related. Our task at this stage was to decide whether each group of competencies was potentially relevant to our purposes. This was a simple task that resulted in perfect agreement.

We also found substantial agreement that 152 of the individual competence items of the CBT framework were relevant for HBCCF. This level of agreement does not simply reflect shared working of the two coders as it was conducted independently prior to discussion and subsequent stages in developing HBCCF did not add further competences that might have been found in the CBT framework.

However, the competencies identified in this way mainly covered the competencies to reach the point of delivering the active ingredients of the intervention but did not address competency to deliver actual BCTs. Thus, it became apparent that a richer source of active behaviour change methods was required.

The competencies derived from the CBT framework were classified into two domains. The first, foundation competencies, addresses the knowledge and skills that might be necessary for anyone delivering a person-to-person service, for example including surgeons and lawyers, as they deal with the legal ethical and professional frameworks and with the effective, non-discriminatory communication needed to engage the client and continue to work with them. The second, behaviour change competencies, deals with knowledge and skills relevant to delivering services for personal change, including assessment and intervention planning before delivery of active ingredients, i.e. BCTs; some of these competencies would be relevant for any intervention designed to enable the recipient to make change while others are more specific to behaviour change. The foundation and behaviour change competencies were modified in the third phase of development by feedback from potential users but we would expect this to be a continuing process of modification, including additions and removals.

In summary, our approach was to begin with expert consensus and to offer a framework based on this consensus. This consensus-based framework should be explored and tested by the wider academic community as well as by those tasked with developing, delivering, and assessing training. We recognise that there is always a decision to be made as to at what point do you stop any development process and publish the outcomes of that development, in this case the HBCCF. Our approach was to be advised by our stakeholders as to when the framework was at a stage of being credible, acceptable, and feasible to use by those using it. At that stage, the government published the framework for use via their website. Here, our aim is to describe the development of the HBCCF and to make it available for wider scrutiny and evaluation.

The HBCC was developed using an early version of the BCT taxonomy that was later published as the BCTTv1 [9]. BCTv1 was considered suitable as it is widely used for describing the active content of behaviour change interventions. We also anticipate that the HBCCF will continue to require updating as that taxonomy matures and is informed by additional evidence. We anticipate improvements to HBCCF with continued use.

Study 2 illustrates how the competencies required for an intervention can be specified leading to the appointment of staff with the required skills for the specific intervention. In a research context, the HBCCF allows more precise description of the training and skills of those delivering an intervention as required by TIDieR under the heading ‘WHO’ [12] and may also assist in explaining differences in effectiveness between different health behaviour change interventions. That said, the modest interrater agreement obtained in the current study indicates that the terminology used to describe competencies could be improved. The ability to code reliably the behaviour change content of behaviour change interventions has benefitted enormously from the publication of the BCTTv1, which provides a shared and agreed terminology for BCTs. This shared language enables the BCT content of behaviour change interventions to be described with precision and for that content to be recognised reliably. The development of an equivalent agreed terminology for competencies to deliver behaviour change interventions would undoubtedly be useful.

Other related competency frameworks have been developed. The NHS Yorkshire and Humber ‘Prevention and Lifestyle Behaviour Change’ framework [30] describes competencies for a wide range of lifestyle change but only a small part of this framework addresses interventions to change behaviour. Unfortunately, the methodology for the development of the framework is not described in detail; however, many of the items included overlap with the HBCCF. The competence framework for psychological interventions with people with persistent physical health conditions [31] aims to define competencies for work with a more restricted population, dealing with competencies in working with people with long-term conditions. As a result, it has some overlap insofar as that work involves behaviour change but it does not address change in people without an ongoing clinical condition. It was developed by discussion and consensus rather than by the independent content analysis and coding methods used to develop the HBCCF. However, its development benefitted from the inclusion of individuals from a variety of disciplines working with people with long-term conditions, including practitioners and researchers from general practice, secondary and tertiary referral, academia, and those responsible for the training and development of the NHS workforce. It identifies 31 competency topics organised into six competency domains, plus one domain that specifies specific interventions for people with long term conditions (examples of six CBT-based interventions and one psychodynamic interpersonal therapy are provided). Neither of these two frameworks was developed with reference to the delivery of the active component of behaviour change interventions, namely BCTs.

More recently, Public Health England, the national body responsible for public health in England, has been developing a behaviour change development framework (https://behaviourchange.hee.nhs.uk/) for use in training and in planning developments in health and social care. This framework has incorporated much of the HBCCF but additionally has classified competences according to the different levels of competence that might be required. For example, the lowest level (‘very brief interventions for service users with a primarily administrative need’) includes ‘initiate a discussion about health behaviours’ which is similar to HBCCF foundation competence F5.1 ‘ability to initiate a discussion about potential health behaviour problems’, the next level (‘brief and extended interventions for service users with a specific health or social care need’) includes ‘agree goals for the interventions and ensure they are realistic, attainable, timely and measurable’) comparable with HBCCF behaviour change competency domain BC4 ‘ability to agree goals for the intervention’, and the top-level (‘high-intensity interventions for service users with primarily complex behaviour related needs’) includes ‘adapt interventions in response to service user feedback’ which is similar to foundation domain competency F8 ‘capacity to adapt intervention to client feedback’. At an early stage, HBCCF competences were classified into different levels of intensity of the intervention (http://www.healthscotland.com/uploads/documents/4877-Health_behaviour_change_competency_framework.pdf) *but this should be repeated using the improved definitions of intervention intensity developed by Public Health England.*

Like the NHS Yorkshire and Humber framework, the HBCCF was created to meet the needs of health service delivery. The HBCCF was developed for the Health Directorate of the Scottish Government. The urgent need for this framework was demonstrated by the willingness of policy-makers, training designers, and practitioners to engage in the consultation and feedback processes. The HBCCF has been used widely to develop and implement training on health behaviour change for health professionals across the UK. For example, the utility of competency frameworks, with the HBCCF as the exemplar framework, to structure and evaluate staff training to ensure their competence to deliver behaviour change interventions has been recognised in national guidance [32]. The HBCCF is also currently being used by the National Health Service Education Scotland to develop a behaviour change training programme for NHS staff and is being used by the Scottish Diabetes Group to improve the consultation and behaviour change skills of health professionals working in the area of diabetes. The use of the framework at this stage by training leads simply demonstrates the urgent need for such a framework. The HBCCF was found to be useful and, crucially, was available for use when the NHS Education Scotland needed such a framework. Ideally, more development would have been beneficial but in practical contexts an adequate framework is better than no framework and the use of an explicit framework allows for future improvements in a cumulative manner. The HBCCF also provided an informative framework during the development of a competency framework for psychological interventions in physical health [31].

In planning further work, we anticipated that the HBCCF might be useful in self-assessment by practitioners deciding on training they should undertake or employment they should seek. We are therefore working on an online self-assessment of HBCCF competencies. In addition, we recognise that interventions may require different levels of competence and that health professionals from different disciplines or at different stages in their training may require a different level of competence to deliver behaviour change interventions in their work context. We are therefore examining competences at different levels and stages of professional development.

## Conclusions

A framework for describing the competencies required to deliver health behaviour change is of considerable importance in both research and practical contexts to ensure that intervention delivery is both adequate and reportable. We describe the pragmatic development of the HBCCF for behaviour change interventions delivered person-to-person based on an existing framework (CBT competency framework) and with reference to an early version of the BCTTv1, amplified by examination of relevant research and application literatures. The two domains (foundation and behaviour change) describe a comprehensive set of competencies which have proved useful in practical situations and are illustrated here for smoking cessation interventions. In sum, the HBCCF is a usable, useful framework but one which will be adapted with use.

## Electronic Supplementary Material

ESM 1 (PDF 716 kb)

## Data Availability

All data generated or analysed during this study are included in this published article and its supplementary information files.

## Footnotes

1 At the time the HBCCF was being developed the BCTTv1 was not yet finalised and published. Therefore, a preliminary version of the taxonomy was used to inform judgements about the relevance of competencies to deliver behaviour change interventions, an important active ingredient of which is BCTs.

2 The HBCCF published by the NHS Scotland contains an early version of the taxonomy of BCTs. We presented the BCTs to the NHS Scotland in the form of a 3rd domain to be considered alongside the two competency domains because BCTs are the active components of behaviour change interventions that staff need to be competent to deliver.

## Abbreviations

CBT: cognitive behaviour therapy
BCTs: behaviour change techniques
BCTTv1: Behavior Change Techniques Taxonomy Version 1
HBCCF: Health Behaviour Change Competency Framework
KSF: Knowledge and Skills Framework
NHS: National Health Service
