# Supplementary material for: What Competences Are Required to Deliver Person-Person Behaviour Change Interventions: Development of a Health Behaviour Change Competency Framework
Source: Int J Behav Med. 2020 Jul 20;28(3):308–17. doi: 10.1007/s12529-020-09920-6 (PMC8121720; doi:10.1007/s12529-020-09920-6)
Supplement: Supplementary file 1 — (PDF 716 kb) [file 12529_2020_9920_MOESM1_ESM.pdf]

Electronic Supplementary Material

Three tables are provided. Table 1 (pg 1), Table 2, (pg 17) and Table 3 (pg 24)

Supplementary Table 1

Table 1 has two columns.
Column 1 describes the individual competences within each competency topic, within the foundation and behaviour change competency domains.
Column 2 indicates the equivalent competency from the Knowledge and Skills Framework used by the NHS. The KSF dimension, sub-dimension and level of competency are listed for each HBCC competency. The KSF mapping is described in the following sequence: dimension code; sub-dimension number; level of competency
For example HWB4L3 denotes KSF Health and Wellbeing Dimension (HWB), sub-dimension 4 (*enablement to address health and wellbeing needs*), level of competence 3.
The KSF dimensions are coded in the tables as follows:

- C = Core dimension;
- HWB = Health and Wellbeing dimension;
- IK = Information and Knowledge dimension.

Foundation Competency Domain

F1. Professional and ethical guidelines

	**KSF**
1. Knowledge of national and local codes of practice which apply to all staff involved in the delivery of healthcare, as well as any codes of practice which apply to the health professional as a member of a specific profession	C5L1
2. Knowledge of legislation relevant to areas of professional practice in which the health professional is engaged (specifically including the Human Rights Act, Data Protection Act)	C5L1
3. Knowledge of relevant codes of professional and ethical conduct and practice in order to apply the general principles embodied in these codes to each piece of work being undertaken, in the areas of:	C5L1
3.1. obtaining informed consent for interventions from clients	C1L3<br>C5L1
3.2. maintaining confidentiality, and knowing the conditions under which confidentiality can be breached	C1L1<br>C5L1

3.3. keeping accurate records	C1L1<br>C5L1<br>IK2L1
3.4. safeguarding the client's interests when co-working with other professionals as part of a team, including good practice regarding interworker/inter-professional communication	C1L3<br>C5L1
3.5. competence to practice, and maintaining competent practice through appropriate training/professional development	C2L3<br>C5L1
3.6. recognition of the limits of competence and taking action to enhance practice through appropriate training/professional development	C2L1<br>C5L1
3.7. protecting clients from actual or potential harm from professional malpractice by colleagues by instituting action in accordance with national and professional guidance	C5L3<br>HWB3L1
4. Maintaining appropriate standards of personal conduct for self:	C5L1
4.1. a capacity to recognise any potential problems in relation to power and "dual relationships" with clients, and to desist absolutely from any abuses in these areas	C5L1
4.2. recognising when personal impairment could influence fitness to practice, and taking appropriate action (e.g. seeking personal and professional support and/or desisting from practice)	C5L1
5. Knowledge of and appropriate referral to other agencies when required	C5L1
6. Identifying problems or opportunities for improving any aspect of service (including from client feedback)	C4L1
7. Reporting appropriately problems or opportunities for improving any aspect of service	C4L1

F2. Supervision

	**KSF**
1. Knowledge that the primary purpose of supervision and learning is to enhance the quality of the intervention clients receive	C2L1
2. An ability to work collaboratively with the supervisor	C1L2<br>C2L2
2.1. An ability to work with the supervisor in order to generate an explicit agreement about the parameters of supervision (e.g. setting an agenda, being clear about the respective roles of supervisor and supervisee, the goals of supervision and any contracts which specify these factors)	C1L2<br>C2L2

2.2. An ability to help the supervisor be aware of your current state of competence and your training needs	C1L2<br>C2L2
2.3. An ability to present an honest and open account of client work undertaken	C1L2<br>C2L2
2.4. An ability to discuss client work with the supervisor as an active and engaged participant, without becoming passive or avoidant, or defensive or aggressive	C1L2<br>C2L2
3. A capacity for active learning	C2L2
3.1. An ability to act on suggestions regarding relevant reading made by the supervisor, and to incorporate this material into work with clients	C2L2
3.2. An ability to take the initiative in relation to learning, by identifying relevant papers, books, or web-based resources based on (but independent of) supervisor suggestions, and to incorporate this material into work with clients	C2L2<br>IK3L1
4. An ability to reflect on the quality of supervision as a whole, and (in accordance with national and professional guidelines) to seek advice from others where:	C1L3<br>C2L3
4.1. there is concern that supervision is below an acceptable standard	C1L3<br>C2L3
4.2. where the supervisor's recommendations deviate from acceptable practice	C1L3<br>C2L3
4.3. where the supervisor's actions breach national and professional guidance (e.g. abuses of power and/or attempts to create dual (sexual) relationships)	C1L3<br>C2L3

F3. Knowledge of and ability to work with difference

	**KSF**
1. Knowledge of the potential significance for practice of social and cultural difference, across a range of domains, including:	C1L2<br>C6L2
1.1.ethnicity	C1L2<br>C6L2
1.2.culture	C1L2<br>C6L2
1.3.education	C1L2<br>C6L2
1.4.deprivation level/SES	C1L2<br>C6L2

1.5.religion	C1L2<br>C6L2
1.6.gender	C1L2<br>C6L2
1.7.age	C1L2<br>C6L2
1.8.disability	C1L2<br>C6L2
1.9.sexual orientation	C1L2<br>C6L2
2. Knowledge of the relevance and potential impact of social and cultural difference on the **effectiveness** and *acceptability* of an intervention for all clients	*C6L2*<br>**C6L3**<br>HWB7L2
3. Ability to make appropriate adjustments to the intervention, with the aim of maximising its potential benefit to the client where social and cultural difference impacts on the accessibility of intervention	HWB7L3

F4. Ability to communicate and work with different individuals, groups and communities

	**KSF**
1. Ability to work and communicate effectively with:	C1L2
1.1. individuals	C1L2
1.2. groups	C1L2
1.3. significant others (including: spouses, partners, relative, families, other social groups)	C1L2
1.4. people in own and other agencies	C1L2

F5. Ability to engage client

	**KSF**
1. Ability to initiate a discussion about potential health behaviour problems	C1L3
1.1. ability to create an environment suitable for frank, confidential discussion	C1L2
1.2. ability to explain why the client's health behaviour is of interest	C1L3

1.3. ability to initiate discussions about risky health behaviour and respond to clients who express concerns about their health behaviour	C1L3
2. While maintaining professional boundaries, an ability to show appropriate levels of warmth, concern, confidence and genuineness, matched to client need	C1L3
3. An ability to engender trust	C1L2
4. An ability to develop rapport	C1L2
5. An ability to adapt personal style so that it meshes with that of the client	C1L3
6. An ability to adjust the level and structure of the session to the client's needs	C1L3
7. An ability to convey an appropriate level of confidence and competence	C1L2
8. An ability to avoid negative interpersonal behaviours (such as impatience, aloofness, or insincerity)	C1L2

F6. Ability to work with groups of clients

	**KSF**
1. Knowledge of and ability to draw on this knowledge in practice of professional and ethical factors in relation to working with groups	C1L3
2. Ability to apply professional and ethical standards when working with groups	C1L3
3. Ability to ensure the group adopts appropriate ethical standards, e.g. confidentiality and respect	C1L3
4. Ability to engage the group (see ability to engage client above)	C1L3
5. Ability to report on missing members	C1L3
6. Ability to encourage group discussions/didactic presentations	C1L3
7. Ability to communicate rules governing the group	C1L3
8. Ability to emphasis that each individual has a responsibility to the group	C1L3
9. Ability to establish a closed group	C1L3
10. Ability to communicate group member identities	C1L3
11. Ability to **communicate** and maintain rules of group behaviour	**C1L3**

F7. Ability to foster and maintain a good intervention alliance, and to grasp the client's perspective*

Knowledge of the concept of the intervention alliance

	**KSF**
1. Knowledge that the therapeutic alliance is usually seen as having three components:	HWB7L3
1.1. the relationship or bond between health professional and client	HWB7L3
1.2. consensus between the health professional and client regarding the goals of intervention	HWB7L3
1.3. consensus between the health professional and client regarding the techniques/methods employed in the intervention	HWB7L3
1.4. An ability to draw on knowledge that all three components contribute to the maintenance of the alliance	HWB7L3

Capacity to develop and maintain the alliance

	**KSF**
2. An ability to listen to the client's concerns in a manner which is nonjudgmental, supportive and sensitive, and which conveys a comfortable attitude when the client describes their behaviour and experience	C1L3
3. An ability to ensure that the client is clear about the rationale for the intervention being offered	C1L3<br>HWB7L3
4. An ability to gauge whether the client: a) understands the rationale for the intervention, b) has questions about it, or c) is skeptical about the rationale, and to respond to these concerns openly and non-defensively	C1L3<br>HWB7L3
5. An ability to help the client express any concerns or doubts they have about the intervention and/or the health professional, especially where this relates to mistrust or skepticism	C1L3<br>HWB7L3
6. An ability to respond appropriately to interventions in response to disagreements about tasks and goals:	C1L3<br>HWB7L3
6.1. An ability to check that the client is clear about the rationale for the intervention and to review this with them and/or clarify any misunderstandings	C1L3<br>HWB7L3
6.2. An ability to help clients understand the rationale for the intervention through using/drawing attention to concrete examples	C1L3<br>HWB7L3
7. An ability to use humour judiciously, understanding how it can be used as an aid to help clients (e.g. to normalise the client's experience or to reduce tension), but also recognising its risks (e.g. of invalidating the client's feelings, acting as a distraction to/ avoidance of feelings, or creating "boundary violations")	C1L3
8. An ability to respond to client's humour in a manner that is congruent with its intent, and responsive to any implied meanings	C1L3

F8. Capacity to adapt interventions in response to client feedback

	KSF
1. An ability to accommodate issues the client raises explicitly or implicitly, or which become apparent as part of the process of the intervention:	HWB4L3<br>HWB7L3
2. An ability to respond to, and openly to discuss, explicit feedback from the client which expresses concerns about important aspects of the intervention	C1L3<br>HWB4L3
3. An ability to detect and respond to implicit feedback which indicates that the client has concerns about important aspects of the intervention (e.g. as indicated by non-verbal behaviour, verbal comments or significant shifts in responsiveness)	HWB7L1

F9. Ability to Manage Expectations of the Intervention

	KSF
1. Ability to communicate the frequency and duration of consultations	C1L3<br>HWB7L3
2. Ability to communicate what is expected of client between consultations	C1L3<br>HWB7L3
3. Ability to manage endings	C1L3<br>HWB7L3
3.3. An ability to signal the ending of the intervention at appropriate points during the intervention (e.g. when agreeing the intervention contract, and especially as the intervention draws to close) in a way which acknowledges the potential importance of this transition for the client	C1L3<br>HWB7L3
3.4. An ability to review the work undertaken together	C1L3<br>HWB7L3
3.5. An ability to say goodbye	C1L3<br>HWB7L3

F10. Ability to deliver information

	KSF
1. An ability to deliver information in a manner that can be understood by the client	C1L3
2. An ability to give instruction in a manner client can follow	C1L3
3. Ability to give advice in a manner that enables client to choose whether or not to take advice	C1L3
4. Ability to give advice about additional resources (including medication) and support relevant to the health behaviour problem	C1L3

	HWB7L3

F11. Capacity to structure consultations and maintain appropriate pacing

	KSF
1. An ability to maintain adherence to an agreed agenda and to 'pace' the consultation in a manner which ensures that all agreed items can be given appropriate attention (i.e. ensuring that significant issues are not rushed)	HWB7L3
2. An ability to balance the need to maintain adherence and pacing while being appropriately responsive to client need:	HWB7L3
2.1. An ability to structure the session in a manner which is congruent with specific issues (e.g. the client's capacity to concentrate)	HWB7L3
3. An ability to balance the need to maintain an appropriate pace v following up important issues raised by the client:	HWB7L3
3.1. An ability to use professional judgment to decide when issues needs to be pursued and when they could act to divert attention from the primary (and agreed) focus of the intervention	HWB7L3

F12. Ability to recognise barriers to and facilitators of implementing interventions

	KSF
1. Ability to recognize barriers to and facilitators of implementing interventions at the level of:	C4L3
1.1. Organisational barriers and facilitators:	C4L3
1.1.1. availability of time and resources to enable implementation of interventions	C4L3
1.1.2. organizational attitudes	C4L3
1.1.3. provision of supervision and ongoing support to practice	C4L3
1.2. Individual health professional barriers and facilitators:	C5L3
1.2.1. own beliefs and attitudes to health behavior and behaviour change	C5L3
1.2.2. competences required to implement interventions	C5L3
1.2.3. adequate post in which to deliver interventions	C4L3<br>C5L3
1.3. Client barriers and facilitators:	HWB6L3
1.3.1. beliefs, attitudes, health condition	HWB6L3
1.3.2. social and physical environment	HWB6L3

BC1. Knowledge of Health behaviour and health behaviour problems

	KSF
Knowledge of:	
1. Common health behaviour problems during assessment and when carrying out interventions, including knowledge of national guidelines for health behaviours, e.g. alcohol consumption limits, recommended physical activity levels etc	HWB6L3<br>HWB7L3
2. Factors associated with the development and maintenance of health behaviours	HWB7L3
3. The usual patterns of health behaviour problems	HWB7L3
4. The ways in which health behaviour problems can impact on health and functioning	HWB7L3
5. The usual knowledge and misinformation that people may have about health behaviour problems	HWB7L3
6. Main terms and concepts used in epidemiology and the basis of calculations related to these terms	HWB7L3
7. Different models, principles and approaches to managing risk	HWB7L3
8. Different models, principles and approaches to preventing risk and threats to population health	HWB7L3
9. Different models, principles and approaches to improving the health of individuals	HWB7L3

BC2. Ability to undertake a generic assessment

	KSF
1. An ability to obtain a general idea of the nature of the client's problem	C1L3<br>HWB6L3
2. An ability to elicit information regarding health behaviour problems and diagnosis	C1L3<br>HWB6L3
3. Ability to elicit information about past history and present life situation	C1L3<br>HWB6L3
4. Ability to elicit information about behavioural and other risk factors for disease	C1L3<br>HWB6L3
5. Ability screen client for suitability for group based support where appropriate	C1L3<br>HWB6L3
6. Ability to screen client for suitability for behaviour change or referral to specialist help	C1L3

	HWB6L3
7. An ability to gauge the client's motivation for a behaviour change intervention	C1L3<br>HWB6L3

BC3. Knowledge of a model of behaviour change and the ability to understand and employ the model in practice

	**KSF**
1. Knowledge of the factors common to all behaviour change models and methods:	HWB6L3
1.1. Support factors:	HWB6L3
1.1.1.a positive working relationship between health professional and client characterised by warmth, respect, acceptance and empathy, and trust	HWB6L3
1.1.2.the active participation of the client	HWB6L3
1.1.3.health professional expertise	HWB6L3
1.1.4.opportunities for the client to discuss matters of concern	HWB6L3
1.2. Learning factors:	HWB6L3
1.1.1.information	HWB6L3
1.1.2.advice	HWB6L3
1.1.3.feedback	HWB6L3
1.1.4.changing expectations of personal effectiveness	HWB6L3
1.1.5.assimilation of problematic experiences	HWB6L3
1.3. Action factors:	HWB6L3
1.3.1.behavioural regulation	HWB6L3
1.3.2.cognitive mastery	HWB6L3
1.3.3.experience of successful coping	HWB6L3
2. Knowledge of the principles which underlie the intervention being applied, using this to inform the application of the specific techniques which characterise the model	HWB6L3

3. Knowledge of the principles of the behaviour change model in order to implement the intervention in a manner which is flexible and responsive to client need, but which also ensures that all relevant components are included	HWB6L3
4. Knowledge of the evidence base for the effectiveness of behaviour change models	HWB6L3

BC4. Ability to agree goals for the intervention

1. An ability to help the client generate their own goals for the intervention, and to reach a shared agreement about these, by helping them:	HWB4L3<br>HWB7L3
1.1. to translate vague/abstract goals into specific and concrete goals	HWB4L3
1.2. to identify goals which will be subjectively and objectively observable and potentially measurable (i.e. to ensure that if change takes place it will be noticeable to the client and to others)	HWB4L3
2. An ability to work with the client to ensure that goals are realistic, attainable and timely	HWB4L3

BC5. Capacity to implement behaviour change models in a flexible but coherent manner

	**KSF**
1. An ability to implement a model of behaviour change in a manner which is flexible and which is responsive to the issues the client raises, but which also ensures that all relevant components of the model are included	HWB7L<br>3
2. An ability to maintain adherence to a model without inappropriate switching between modalities in response to minor difficulties (i.e. difficulties which can be readily accommodated by the model being applied)	HWB6L<br>3

BC6. Capacity to select and skillfully to apply the most appropriate behaviour change intervention method

1. An ability draw on knowledge of behaviour change models and methods and on professional experience in order to select from the complete range of behaviour change techniques, and skillfully apply them in a manner which is:	HWB7L3<br>HWB6L3
1.1. matched to the needs and capacities of the client	HWB7L3<br>HWB6L3
1.2. applied at the appropriate level of progression in the process of behaviour change	HWB7L3<br>HWB6L3

BC7. Capacity to implement behaviour change in a manner consonant with its underlying philosophy

Basic orientation

1. An ability to base all health professional/client contact and conduct on a perspective which sees the world, including interactions with the health professional, from the perspective of the client's beliefs and abilities	C1L3<br>HWB7L3

Capacity to form and maintain a collaborative stance

2.	A capacity to form a collaborative relationship with the client, based on an active stance which focuses on enabling the client and the health professional to work as a team	HWB4L3 HWB7L3
3.	An ability to balance the need to structure consultations as against the need to allow the client to make choices and take responsibility	HWB7L3
4.	An ability to avoid implementing behaviour change in a manner which becomes didactic, directive, intellectual or controlling	HWB4L3 HWB7L3

Maintaining a problem solving perspective

5.	An ability to avoid seeing the client themselves as a problem, but to maintain a problem-solving approach to the client's health behaviour problems	HWB4L3 HWB7L3
6.	An ability to maintain a problem-solving attitude in the face of difficulties and frustrations	HWB4L3 HWB7L3

BC8. Ability to structure consultations

	**KSF**
1. An ability to structure consultations	HWB7L3
2. An ability to share responsibility for consultation structure & content	HWB7L3
2.1. An ability to be appropriately structured (especially in the initial stages of the intervention), but also to avoid becoming inappropriately didactic	HWB7L3
3. An ability to agree and adhere to an agreed agenda	HWB7L3
3.1. An ability to work collaboratively with the client to set a mutually agreed agenda at the start of each consultation	HWB7L3
3.2. An ability to set an agenda that is:	HWB7L3
3.2.1. appropriate to the client's health behaviour problem	HWB7L3
3.2.2. appropriate for the level of progression in the process of behaviour change	HWB7L3
3.2.3. consistent with the shared understanding of the client's problem	HWB7L3
3.2.4. an ability to prioritise agenda items, and set an agenda which fits with the time available	HWB7L3
3.2.5. an ability to adhere to the agenda	HWB7L3
3.3. An ability to pace the consultation and use time efficiently	HWB7L3
3.3.1. an ability to 'time manage' the consultation in relation to the agenda	HWB7L3
3.3.2. an ability to pace the consultation in relation to the client's needs and learning speed	HWB7L3
4. An ability to make and review action plans	HWB4L3
4.1. Ability to plan action plans	HWB4L3
4.1.1. an ability to work with the client to agree appropriate and manageable action plans	HWB4L3
4.1.2. an ability to work with client to identify strategies which will help ensure that action plans are carried out	HWB4L3

4.2. Ability to review action plans	HWB6L3
4.2.1. an ability to ensure that action plans that the client has undertaken are carefully discussed and reviewed with them in the next session, with the aim of helping them identify what they have learned	HWB6L3
4.2.2. An ability to help clients appraise the outcomes of action plans:	HWB6L3
4.2.2.1. when outcomes are in line with the prior expectations of the health professional and client	HWB6L3
4.2.2.2. when there is a different outcome from that which has been predicted	HWB6L3
4.2.3. an ability to integrate learning from action plans into the session, and to build on this learning in identifying further action plans	HWB4L3<br>HWB6L3
5. An ability to use summaries and feedback to structure the consultation	HWB4L3
5.1. An ability to structure the consultation by regularly giving feedback to the client, and by eliciting regular feedback from the client	HWB6L3
5.2. An ability to elicit and respond both to verbal and non-verbal feedback from the client throughout the consultation (i.e. to take into account explicit statements made by the client and their emotional reactions)	HWB6L3
5.3. An ability to give verbal feedback to the client throughout the session, by offering 'capsule' summaries and by 'chunking' important (salient) information and/or topics	HWB4L3<br>HWB6L3
5.4. An ability to invite summaries from the client (to check that the health professional understands the client's health behaviour problems and that the client understands what the health professional is saying)	C1L3<br>HBW4L3
5.5. An ability to offer summaries at the start of consultations (e.g. **a review of prior work**) and at the end of the session (*covering the main points of the consultation*)	*HWB4L3*<br>**HWB6L3**

BC9. Ability to use measures and self-monitoring to guide behaviour change interventions and to monitor outcome

	**KSF**
1. Knowledge of commonly used questionnaires and rating scales	HWB6L3
2. Ability to select and interpret measures:	HWB6L3
2.1. Ability to select measures relevant to the client's health behaviour problem	HWB6L3
2.2. An ability to interpret measures:	HWB6L3
2.2.1. An ability to interpret scores on standard measures relevant to client's health behaviour problem	HWB6L3
2.2.2. An ability to draw on knowledge regarding the interpretation of measures (e.g. basic principles of test construction, norms and clinical cut-offs, reliability, validity, factors which could influence (and potentially invalidate) test results)	HWB6L3
2.2.3. An ability to be aware of the ways in which the reactivity of measures and self-monitoring procedures can bias client report	HWB6L3
3. Knowledge of self-monitoring:	HWB6L3

3.1. An ability to draw on knowledge of self-monitoring forms developed for use in specific interventions (as published in articles, textbooks, manuals or web-based resources)	HWB6L3
3.2. Knowledge of the advantages of using self-monitoring (to gain a more accurate concurrent description of behaviours (rather than relying on recall), to help adapt the intervention in relation to client progress, and to provide the client with feedback about their progress)	HWB6L3
3.3. Knowledge of the role of self-monitoring in behaviour change (a means of helping the client to become an active, collaborative participant in their own behaviour change by identifying and appraising how they react to events (in terms of their own physiological reactions, behaviours, feelings and cognitions))	HWB6L3
3.4. An ability to draw on knowledge of measurement to ensure that procedures for self-monitoring are relevant (i.e. related to the question being asked), valid (measuring what is intended to be measured) and reliable (i.e. reasonably consistent with how things actually are)	HWB6L3
4. Ability to integrate measures into the intervention:	HWB6L3
4.1. An ability to use and to interpret relevant measures at appropriate points throughout the intervention, with the aim of establishing both a baseline and indications of progress	HWB6L3
4.2. An ability to share information gleaned from measures with the client, with the aim of giving them feedback about progress	HWB6L3
4.3. An ability to establish an appropriate schedule for the administration of measures, avoiding over-testing, but also aiming to collect data at more than one time point	HWB6L3
5. Ability to help clients use self-monitoring procedures:	HWB6L3
5.1. An ability to construct individualised self-monitoring forms, or to adapt 'standard' self-monitoring forms, in order to ensure that monitoring is relevant to the client	HWB6L3
5.2. An ability to work with the client to ensure that measures of the targeted problem are meaningful to the client (i.e. are chosen to reflect the client's perceptions of the problem or issue)	HWB6L3
5.3. An ability to ensure that self-monitoring includes targets which are clearly defined and detailed, in order that they can be monitored/recorded reliably	HWB6L3
5.4. An ability to ensure that the client understands how to use self-monitoring forms (usually by going through a worked example during the consultation)	HWB6L3
6. Ability to integrate self-monitoring into the intervention	HWB6L3
6.1. An ability to ensure that self-monitoring is integrated into the intervention, both in the consultation and as part of action plans, ensuring that the agenda for the consultation includes regular and consistent review of self-monitoring forms	HWB6L3
6.2. An ability to guide and to adapt the intervention in the light of information from self-monitoring	HWB6L3

BC10. Ability to carry out health behaviour problem solving

	KSF
1. An ability to identify health behaviour problems facing the client, which may be appropriate for a problem solving approach	HWB4L3
2. An ability to explain the rationale for problem-solving to the client	C1L3<br>HWB7L3
3. An ability to help the client to select problems, usually on the basis that problems are relevant for the client and are ones for which achievable goals can be set	HWB4L3
4. An ability to help the client specify the problem(s), and to break down larger problems into smaller (more manageable) parts	HWB4L3
5. An ability to identify achievable goals with the client, bearing in mind the client's resources and likely obstacles	HWB4L3
6. An ability to help the client generate ("brainstorm") possible solutions	HWB4L3
7. An ability to help the client select a preferred solution	HWB4L3
8. An ability to help the client plan and implement preferred solutions	HWB4L3
9. An ability to help the client evaluate the outcome of implementation, whether positive or negative	HWB6L3

BC11. Capacity to manage obstacles to carrying out behaviour change

	KSF
1. An ability to work collaboratively with client behaviours that are potentially counter-productive – for example, clients who:	C1L3
1.1. find it difficult to talk	HWB6L3
1.2. tend to talk too much and/or find it hard to stay focused	HWB6L3
1.3. invent or distort material	HWB6L3
1.4. are persistently late	HWB6L3

BC12. Ability to end the intervention in a planned manner and to plan for long-term maintenance of gains after intervention ends

	KSF
1. An ability to terminate the intervention in a manner which is planned, and to signal plans for termination at appropriate points throughout the intervention	HWB7L3
2. An ability to plan for maintenance of behaviour change after the end of the intervention:	HWB7L3
2.1. An ability to help clients identify and elaborate their concerns about termination (e.g. worry that that they need support to manage on their own, or that they will relapse)	HWB7L3

2.2. ability to help clients identify other resources that might help them maintain their behaviour change (e.g. weightwatchers, websites, gym membership)	HWB7L3

Supplementary Table 2

Table 2 below details (in alphabetical order) behaviour change techniques as they were described in an early version of the BCT taxonomy and as used during the development of the HBCCF together with the KSF coding.

Behaviour Change Techniques

**Name of BCT**	**Behaviour Change Technique Description**	**KSF**
Action Planning	Make a detailed plan of what the client will do including, as a minimum, when, and where to act	HWB4L 3
Anger Control Training	A combination of techniques that are used to enable the client to control anger	HWB4L 3
Antecedents and consequences	Record antecedents and consequences of behaviour (e.g. social and environmental situations and events, emotions, cognitions)	HWB4L 3
Anticipated regret	Induce expectations of future regret about non-performance of behaviour	HWB4L 3
Assertion Training	A combination of techniques used to teach client interpersonal communication to help them express emotions, opinions, and preferences (positive and negative) clearly, directly, and in an appropriate manner (e.g. for a client who eats or smokes following interpersonal conflict)	HWB4L 3
Avoidance	Identify and advise client to avoid those particular situations, activities, environments, individuals, things, or subjects of thought or conversation that have anticipated negative consequences	HWB4L 3
Behavioural experiments	Identify and test hypotheses about the behaviour, its causes and consequences, by collecting and interpreting data	HWB4L 3 HWB6L 3
Behavioural rehearsal	Provide or identify opportunities for client to perform behaviour repeatedly	HWB7L 3
Biofeedback	Use an external monitoring device to provide an individual with information regarding his or her physiological state, which many enable voluntary control over autonomic body functions e.g. heart rate feedback in increasing physical activity	HWB6L 3
Chaining	Build up behaviour by starting with final component; gradually add components earlier in sequence	HWB7L 3

Classical Conditioning	Present a neutral stimulus jointly with a stimulus that already elicits a response repeatedly until the neutral stimulus elicits that response (Pavlovian Conditioning) e.g. repeatedly pairing fatty foods with a disliked flavoured sauce	HWB7L3
Cognitive restructuring	Change cognitions about causes and consequences of behaviour	HWB4L3
Comparison	Provide comparative data (cf standard behaviour, person's own past behaviour, others' behaviour)	HWB4L3
Contingent Reward	Identify and provide a contingent valued consequence of the target behaviour if and only if the target behaviour is performed (rewards can include social approval)	HWB7L3
Contract	Generate a contract of agreed performance of target behaviour with at least one other, written and signed or verbal	HWB4L3
Coping planning	Identify and plan ways of overcoming barriers (note, this must include identification of specific barriers e.g. "problem solving how to fit into weekly schedule" would not count)	HWB4L3
Coping strategies	Identify behaviours to be undertaken to avoid or reduce stressors	HWB4L3
Counter-conditioning	Reward client for responding to a stimulus in a manner that is incompatible with their previous response to that stimulus, (e.g. reward client for ordering a soft drink when they first go to the bar rather than an alcoholic drink)	HWB7L3
Covert Conditioning	Provide opportunities for client to imagine performing a desired behaviour in a problematic real-life situation, and to reward himself or herself for mentally engaging in the behaviour. Also called covert behavioural reinforcement	HWB7L3
Covert Sensitization	Provide opportunities for client to imagine performing the undesired behaviour (e.g. overeating) and then imagine an unpleasant consequence (e.g. vomiting)	HWB7L3
Decision-making	Generate alternative courses of action, and pros and cons of each, and weigh them up	HWB4L3
Desensitisation	Identify and provide exposure to threatening experiences	HWB7L3
Differential Reinforcement	Arrange for reinforcement of only selected behaviour (e.g. provide reward of consumption of low fat foods but not consumption of high fat foods)	HWB7L3
Discrepancy assessment:	Highlight nature of discrepancy (direction and amount) between standard, own or others' behaviour (goes beyond simple self-monitoring)	HWB4L3
Discrimination Training	Reward the behaviour in one situation but not in another (e.g. reward for eating sweet foods at mealtimes but not between meals	HWB7L3

Discriminative (learned) cue	Identify an environmental stimulus that has been repeatedly associated with contingent reward for specified behaviour	HWB7L 3
Distraction	Identify alternative focus for client's attention to avoid attention to triggers for problematic behaviour and instruct on using in problematic situations.	HWB4L 3
Emetic Therapy	Provide client with drugs that produce aversive side effects when combined with problem e.g. the use of Antabuse in reducing alcoholism	HWB7L 3
Environmental change	Change the environment in order to facilitate the target behaviour (other than prompts, rewards and punishments, e.g. choice of food provided)	HWB4L 3
Escape Learning	Arrange for the termination of an aversive stimulus The principle is identical to that of negative reinforcement	HWB7L 3
Exposure	Provide systematic confrontation with a feared stimulus, e.g. a period of withdrawal from nicotine for a client who wants to stop smoking, either live or in the imagination (may encompass any of a number of behavioural interventions, including systematic desensitization, flooding, implosive therapy and extinction-based techniques)	HWB7L 3
Extinction	Discontinue reinforcement of target behaviour e.g. reduce amount of social attention to smoking behaviours	HWB7L 3
Fading	Provide a gradual changing of one stimulus to another (often used to transfer stimulus control). Stimuli can be faded out or faded in, e.g. gradually increasing the range of foods offered to a client who is trying to choose healthy foods	HWB7L 3
Fear arousal	Induce negative (aversive) emotional state associated with the behaviour	HWB4L 3
Feedback	Provide feedback of monitored (inc. self-monitored) behaviour	HWB6L 3
Flooding	Expose client directly to a maximum-intensity anxiety-provoking situation or stimulus, either in the imagination or in reality. Flooding techniques aim to reduce anxiety that is interfering with desired behaviour e.g. taking client to a gym to overcome anxiety about engaging in physical activity	HWB7L 3
General information	Provide general information about the behaviour and behaviour change	HWB4L 3
General problem-solving	Engage client in general problem-solving	HWB4L 3
Goal review	Assess extent to which the goal/target behaviour is achieved, identify the factors influencing this and amend goal if appropriate	HWB4L 3

Goal setting	Identify and set a behavioural goal	HWB4L 3
Graded tasks	Set easy tasks to perform, making them increasingly difficult until target behaviour is performed	HWB4L 3
Habit formation	Provide or identify opportunities for client to perform same behaviour in the same context repeatedly	HWB7L 3
Habit reversal	Provide opportunities for repeated rehearsal of a new correct response to a stimulus and stop responding to a previously learned cue, (e.g. client responds to a morning coffee break by eating a piece of fruit instead of a piece of chocolate)	HWB7L 3
Homework	Set homework tasks that repeat or build on work done with client e.g. perform mental rehearsal or other BCTs	HWB4L 3
Imagery	Use planned images (visual, motor, sensory) to implement BCTs (inc. mental rehearsal)	HWB4L 3
Implosive Therapy	Repeatedly encourage client to imagine an anxiety-arousing situation, and to experience anxiety as intensely as possible while doing so. Since there is no actual danger in the situation, the anxiety response is not reinforced and therefore is gradually extinguished	HWB7L 3
Information about the behaviour	Provide information about antecedents or consequences of the behaviour, or connections between them, or behaviour change techniques	HWB4L 3
Instruction	Teach new behaviour required for performance of target behaviour (not as part of graded hierarchy or as part of modelling) (e.g. give clear instructions)	HWB4L 3
Mental rehearsal	Provide opportunities for client to imagine performing the behaviour repeatedly	HWB7L 3
Modelling	Prompt observation of the behaviour of others	HWB4L 3
Motivational interviewing	Elicit self-motivating statements & evaluation of own behaviour to reduce resistance to change	HWB4L 3
Negative punishment	Remove a reward as a consequence of a response. (e.g., subtract money from a prepaid refundable deposit when client smokes a cigarette.	HWB7L 3
Negative reinforcement	Identify and remove an aversive consequence of the behaviour, if and only if the behaviour is performed	HWB7L 3
Noncontingent reinforcement	Provide a reward independently of any particular target behaviour.	HWB7L 3

Omission	Identify and remove a contingent valued consequence, if and only if the behaviour is not performed	HWB7L 3
Overcorrection	When a client exhibits inappropriate behaviour ask the client to repeat the behaviour in an appropriate but exaggerated way	HWB7L 3
Paradoxical Instructions	Instruct the client to do precisely the opposite of what common sense would dictate in order to show the absurdity or self-defeating nature of the client's original intention (e.g. smoking many more cigarettes than the client would normally)	HWB4L 3
Prompt	Identify a stimulus that elicits behaviour (inc. telephone calls or postal reminders designed to prompt the behaviour)	HWB7L 3
Punishment	Identify and provide a contingent aversive consequence, if and only if the behaviour is not performed	HWB7L 3
Rational Emotive Therapy	Teach the client, using a variety of cognitive, emotive and behavioural techniques, to modify and replace self-defeating thoughts to achieve new and more effective ways of feeling and behaving.	HWB4L 3
Reassurance	Encourage client to believe in herself/himself and the possibilities of improvement (e.g. by non-specific supportive comments e.g. 'you'll do fine')	C1L3
Reframing	Encourage client to adopt a different perspective on behaviour in order to change attitude (e.g. by asking how partner would see the behaviour or how they might see it when they were ill or older)	HWB4L 3
Relapse prevention	Identify situations that increase the likelihood of the behaviour not being performed and apply coping strategies to those situations	HWB4L 3
Relaxation	Provide systematic instruction in physical and cognitive strategies to reduce sympathetic arousal, and to increase muscle relaxation and a feeling of calm	HWB4L 3
Response cost	Withdraw a valued commodity from the client as a result of their performing an unwanted behaviour, e.g. loss of access to desired activity e.g. 1hr of TV viewing for each inappropriate behaviour	HWB7L 3
Role play	Provide opportunities for client to perform behaviour in simulated situation	HWB7L 3
Satiation	Expose the client to repeated exposure to a reinforcer e.g. chocolate, in order to reduce its effectiveness of a reinforce	HWB7L 3
Self talk	Make planned self-statements (aloud or silent) to implement behaviour change techniques	HWB4L 3
Self-Monitoring of behaviour	Record the specified behaviour (person has access to recorded data of behavioural performance, e.g. from diary)	HWB4L 3

Shaping	Build up behaviour by initially reinforcing behaviour closest to required behaviour and systematically altering behaviour required to achieve contingent reinforcement	HWB7L 3
Social comparison	Provide opportunities for social comparison, i.e. comparison between self and other people (e.g. contests and group learning)	HWB4L 3
Social Skills Training	Teach effective social interaction in specific situations (e.g. job interviews,), may include techniques such as: behaviour rehearsal, cognitive rehearsal, and assertiveness training	HWB4L 3
Social support (emotional)	Provide &/or id potential sources of empathy and give generalised positive feedback	HWB4L 3
Social support (instrumental)	Provide or arrange for others to perform component tasks of behaviour or tasks that would compete with behaviour (e.g. offering childcare)	HWB4L 3
Social Support (non-specific)	Provide and/or identify sources of non-specific social support	HWB4L 3
Standard	Decide on the target standard of behaviour (specified and observable)	HWB4L 3
Stimulus Generalization	Guide client to spread of effects of learning a behaviour in one situation to other similar situations e.g. exercises learned in gym repeated when wearing informal clothes	HWB7L 3
Stress Inoculation Program	For clients experiencing stress consider using Stress-Inoculation Training (SIT)	HWB4L 3
Systematic desensitisation	Provide graded exposure to increasingly threatening experiences	HWB7L 3
Thinning	Provide a gradual increase in the intermittency of reinforcement, (e.g. gradually increase the time between rewards)	HWB7L 3
Threat	Offer future punishment or removal of reward contingent on performance	HWB7L 3
Time management	Apply action planning to the perceived problem of shortage of time	HWB4L 3
Time Out	Move the client away from the area that is reinforcing the behaviour, e.g. instruct client not to go into the local shop from which they buy their cigarettes	HWB4L 3
Token Economy	Reinforce the desired behaviour by offering tokens that can be exchanged for special foods, television time, passes, or other rewards	HWB7L 3

Verbal persuasion / persuasive communication	Presentation of arguments in favour of the behaviour by a credible source (NB: there must be evidence of presentation of arguments; general pro-behaviour communication does not count)	HWB4L3
Vicarious reinforcement	Prompt observation of the consequences of others' behaviour	HWB4L3

KSF notation as follows: C=core dimension; HWB=health and wellbeing dimension; IK=information and knowledge dimension. The first number refers to the sub-dimension within that dimension. L=level of competence. The second number refers to the level of competence. For example: HWB4L3 denotes KSF Health and Wellbeing Dimension, sub-dimension 4 (enablement to address health and wellbeing needs), level of competence 3.

Supplementary Table 3

Table 3 lists the 23 frequently used BCTs from BCTTv1 (Michie et al 2013). The HBCCF was written whilst the BCTTv1 was in development. As a consequence, there is not a perfect correspondence between the BCTs as described during the development of the HBCCF and those in BCTTv1. Below are listed the 23 most frequently used BCTs and the BCT from Supplementary Table 2 (above) that best corresponds with the BCT as described by BCTTv1. Some of the BCTs from BCTTv1 were not included in version used in the development of the HBCCF and some BCTs in the BCTTv1 describe more than one BCT as described at the time of the development of the HBCCF; these are indicated in the table below.

BCT label in taxonomy (BCTTv1)	BCT label as used during the development of the HBCCF
1. Pharmacological support	Not included[a]
2. Self-monitoring of behaviour	Self-monitoring of behaviour
3. Restructuring of the physical environment	Environmental change
4. Social support (practical)	Social support (instrumental)
5. Behavioural practice/rehearsal	Behavioural rehearsal
6. Problem solving/coping planning	Coping planning<br>*General problem solving*
7. Persuasive argument	Verbal persuasion/persuasive communication
8. Review behaviour goals	Goal review
9. Goal setting (outcome)	Goal setting
10. Prompts/cues	Prompt
11. Demonstration of the behaviour	Instruction (teach new behaviour required for performance of target behaviour)
12. Non-specific encouragement	*Reassurance (encourage client to believe in him/herself and the possibilities of improvement*
13. Review of outcome goal	Goal review
14. Discrepancy between current behaviour	Discrepancy assessment<br>*Comparison*
15. Self-monitoring of outcome of behaviour	*Antecedents and consequences*
16. Health consequences	*General Information (but it is specified as info about the behaviour and behaviour change)*
17. Feedback on behaviour	Feedback
18. Action planning (including implementation intentions)	Action Planning
19. Social support (general)	Social support (non-specific)
20. Social comparison	Social comparison

21. Material reward	*Contingent reward (here this can include social approval)*
22. Incentive	*Contingent reward*<br>*Token Economy*
23. Monitoring outcome of behaviour by others without feedback	Not included[a]

[a]Not included = this BCT from BCTTv1 is not in the BCT list in the HBCCF. *Italics*: BCT listed in the HBCCF that relates to the BCT listed in BCTTv1

Reference

Michie. S, Richardson M, Johnston M, et al. The Behavior Change Technique Taxonomy (v1) of 93 Hierarchically Clustered Techniques: Building an International Consensus for the Reporting of Behavior Change Interventions. *Annals of Behavioral Medicine* 2013;46:81-95
